# Report of Ocular Diseases, at the General Hospital, Fort Pitt, from 21st December, 1822, to 20th December, 1823

**Published:** 1824-09

**Authors:** George R. Melin

**Affiliations:** Assistant Surgeon to the Forces.


THE LONDON
### Medical and Physical Journal.

N° 3 OF VOL. LII.] SEPTEMBER, 1824. [N° 307.

For many fortunate discoveries in medicine, and for the detection of numerous errors, the world is indebted to the rapid circulation of Monthly Journals; and there never existed any work, to which the Faculty, in Europe and America, were under deeper obligations, than to the Medical and Physical Journal of London, now forming a long, but an invaluable, series.—RUSH.

### ORIGINAL COMMUNICATIONS,
### SELECT OBSERVATIONS, &c.

ART. I.—*Report of Ocular Diseases, at the General Hospital, Fort Pitt, from* 21*st December*, 1822, *to* 20*th December*, 1823. By GEORGE R. MELIN, Assistant Surgeon to the Forces.

THE greater number of ophthalmic cases at this hospital, in 1822, were principally admitted from the 13th and 44th Regiments, which were quartered here for some time previous to embarking for India, and were generally recent attacks. This will also explain the reason of the large proportion of men discharged to their regiments, in comparison with former years, when the diseases treated were generally of long standing, and amongst men who were invalided on account of ophthalmia; in which case, the surgeon in charge here cannot well send a man back to his regiment, unless the eyes are restored to a perfectly healthy and natural state; for, if there was the most trifling defect in either eye, he would be liable to be rejected by his surgeon, on joining the regiment.

The cause of the prevalence of the disease in these two regiments, I could never satisfactorily discover: it had existed in both corps for some time, and their respective surgeons were unable to account for it, although they used every means in their power to check its course, and prevent its recurrence. The men were certainly a good deal exposed to changes of atmosphere and temperature, during drill and on night-duty; and many of the cases, especially among the recruits, no doubt were occasioned by this. But then the other troops in the garrison were nearly equally exposed, and did not suffer in the same manner. There was, therefore, too much reason to fear that many of the men, unwilling to embark, had adopted some secret method of producing the inflammation; and in two cases I detected it, but could not succeed in inducing either of the men to disclose the nature of the imposition. I am further confirmed in my suspicion, by Dr. Jones, of the 44th, having told me, since his return to this country, that not a case of ophthalmia occurred during the voyage.

*Ophthalmia.*—In treating of this disease, and detailing the practice I pursued, it will only be requisite to consider it under its acute and chronic forms, without any distinct reference to whether it was accompanied by a purulent secretion or not, as the treatment was the same, and none of the cases were of that severe form commonly called Egyptian.

For some time before I was placed in charge of the ophthalmic division of this establishment, I considered that acute ophthalmia, in general, was treated too actively, and that a mere local inflammation could not require such extensive general depletion as was usually practised and recommended; and, from having witnessed the good effects of a solution of lunar caustic in some cases of gonorrhœa, both in allaying the pain and suppressing the discharge, I was determined, the first opportunity, to try its effects in inflammation of the conjunctiva, a similar membrane to that lining the urethra, and where the only danger attending its use in the latter was not to be apprehended. This opportunity was afforded me shortly after my arrival here; and the first acute cases which were admitted, I pointed out to Dr. Forbes, under whose observation I carried my views into execution, and was soon gratified by their being attended with complete success. The strength of the solution I employed was four grains to the ounce of distilled water, which I dropped into the eyes twice a-day: it excited pain and a sensation of roughness, with an increased flow of tears, for about ten or twenty minutes; after which the eyes felt much relieved, and in a few days the cure was effected.

Since that period I have treated nearly *three hundred cases of acute ophthalmia*, some of them of a severe nature, in a similar manner, without either local or general bleeding, and I have had ample opportunities of proving its efficacy. One very material advantage attending this mode of treatment is, that the inflammation is subdued without leaving any chronic disease, either in the eye-balls or lids; whereas, by the antiphlogistic plan, (though the active inflammation be removed, and the eye saved,) it frequently leaves the vessels in such a debilitated state, that you have a more difficult and tedious disease to contend with, and one which frequently renders vision imperfect. I have received, from several of my professional friends, to whom I communicated the plan of treatment, very satisfactory accounts of its efficacy in their practice; and from Mr. Beard, of the Artillery, who has had much experience in ophthalmic complaints, and who saw many of my cases, I some time ago received a letter, from which the following is an extract:—

"I have used the caustic solution in about thirty cases of acute ophthalmia, and, with the exception of two, (and in those exceptions I was not my own master,) without any auxiliary aid from bleeding, purging, or external application, or even abstraction of the stimuli of light, heat, or meat diet, &c. In the excepted cases, I was ordered to bleed, and pursue otherwise the antiphlogistic plan, on account of the pain over the orbit appearing to have been increased by the solution. In these two cases, the bleeding did immediately remove the pain in the orbit, but the conjunctival inflammation was not at all subdued; and the caustic was again tried, and almost instantly succeeded. I must, in addition, state, that I was not at all sure these two cases were purely and fairly adapted to the caustic treatment. In one of the two men, I suspected he either falsely represented his case, or wilfully aggravated it. In the other, I had reason to believe the iris was concerned. Altogether, I can aver that my success in the caustic plan has been most constant and decided. I have observed that the inflammation of the conjunctiva has not only been rapidly subdued, but that the cure has been remarkably perfect, inasmuch as no elongated or debilitated vessels were left."

In the above extract, there are two circumstances that deserve particular notice: first, it points out the necessity of discriminating between deep-seated and superficial inflammation, and the case alluded to is not the only one in which I have known a similar error to have been committed; and, secondly, the advantage of combining depletion with the use of the solution, in cases where there is severe internal pain. In such cases, I would certainly recommend cupping on the temples; or, if there should exist any increased action of the arterial system, one full bleeding from the arm. But, in the conjunctival inflammation which I have met with here, I have had no reason to employ either; and I am inclined to think that, in the ophthalmia of this country, it can very seldom be required, if treated in its early stage in the manner I have adopted.

This mode of treatment is peculiarly, I think, adapted for regimental practice, as the surgeon sees the disease on its first appearance; and the advantage of employing it thus early is exemplified in the accompanying case, No. 3. In another case, the right eye became affected, while the left was under treatment; and the early use of the solution completely arrested its further progress.

In the severe purulent ophthalmia usually met with in the Mediterranean and warm climates, I should expect that its early exhibition would be attended with great benefit, from the peculiar power it possesses of quickly suppressing the purulent secretion in the ophthalmia of infants, and in the milder forms in which I have seen the disease. Not that I wish to recommend the antiphlogistic remedies to be entirely relinquished, but employed with moderation, in conjunction with the local treatment, where the state of the pulse, or severity of the pain, points out its necessity; as I am convinced, from the many cases of loss of eyes that I have met with and heard of, in which general and local bleeding were carried to a very great extent, that they alone will not save the organ; but, on the contrary, in many of the cases, I am certain that the great abstraction of blood, by reducing the system too low, left the vessels of the part so weak, that they were unable to recover or exert sufficient action to resist the destructive effects of the disease.

The accompanying cases so fully show the nature of the inflammation and the mode of treatment, that I have no more to add on the subject, than to point out the case of William Sparks (No. 4), as a striking illustration, in the same individual, of the difference between the severe antiphlogistic plan of treatment and that which I pursued.

*Chronic Ophthalmia.*—Of this complaint I shall not have occasion to say much, as the day is gone by when the profession was entrapped, by bold and unfounded pretensions to a superior mode of treatment, into an abandonment of the true principles of the science. The knife and scissars are now thrown aside; and even bluestone is, I believe, seldom used with that severity it formerly was. For this improvement and return to reason, the public are indebted to the judgment and persevering exertions of the Medical Board, and of some of its officers.

My treatment has consisted principally in the use of solutions of caustic, varying in strength from four to six grains to the ounce, according to the state of the granulations. If they were very vascular, I employed the weak; if pale and indolent, the stronger; and, in a few cases, where they were remarkably pallid, flabby, and indolent, I employed more active stimulants, —such as solutions of oxymuriate of mercury in the vinum opii. These applications were dropped into the eyes twice a-day, and thus exerted their influence on the opacity and vascularity of the cornea, as well as on the granulations, and the improvement on both was progressive.

Under these gentle remedies, the absorption of the granulations gradually took place, and the vessels of the conjunctiva were restored to a healthy state. I never found it necessary to employ any escharotic; and I am certain that the mild treatment which I have detailed, is attended with more rapid and general benefit and safety to the organ, than that of producing a deep slough, by bluestone or other escharotics, as formerly practised. Long before the granulations or villosity of the lids were removed, the vascularity of the cornea subsided; and, as soon as the lids became smooth, I usually laid aside the solution of the nitras argenti, and, for the remaining opacity of the cornea, I used solutions of the oxymurias hydrargyri, from one to three grains to the ounce of the vinum opii, and other stimulants, according to the susceptibility of the eye to their action; but, when the solution of caustic agreed with the eyes, and the opacity of cornea yielded to its use, I of course continued it, as will be seen by the two accompanying cases.

In a few cases, I found considerable benefit from an alterative course of mercury; but in this and other constitutional treatment, the practitioner must be regulated by the general state of health of the patient; and his success in many of these chronic cases will, in a great measure, depend on the attention and skill which he displays in restoring the system to a healthy state.

This comprises so many and such important points, that it cannot be expected I should enter on them; and I have therefore only given that local treatment, which, combined with attention to the general state of health of the patient, has proved most beneficial in this establishment.

I annex three cases of chronic ophthalmia, which will show the beneficial effects of mild treatment, and that escharotics are not requisite for the cure of granulations.

*Cases of Acute Ophthalmia.* CASE I.—William Wood, 57th Regiment, æt. thirty; admitted 15th March, 1822. Labouring under acute inflammation of both eyes, accompanied with pain, sensation of sand in the eyes, great intolerance of light, and lachrymation, as well as a puriform secretion which was lodged between the inferior lids and eyeballs; the conjunctiva sclerotica extremely vascular; the vessels of a pinkish-red colour, and running at right angles from each other, forming the appearance of a fine net-work over the eyeball. The conjunctiva slightly elevated around the cornea; which is, however, free from vascularity or opacity. The lids extremely vascular, and slightly villous. This attack came on five days ago, while on board the Chapman transport, and gradually increased to the present time.—Instillat sol. argent. nitrat. gr. iv. ad aquæ ℥j.; bis in die amb. oculis. Low diet.

16th.—The pain and sensation of sand considerably abated, and the vascularity diminished. No appearance of chemosis this morning. Bowels rather confined.—Cont. ut antea. Low diet.

17th.—Vascularity diminishing rapidly; no appearance of puriform matter, and all sensation of sand or pain entirely subsided.—Cont. solutio. Low diet.

19th.—Vascularity of the eyeball continues to decline fast; all lachrymation and intolerance of light have ceased; and, to use his own words, he feels the eyes stronger than they have been these two years back.—Cont. solutio. Half diet.

20th.—Vascularity of the eyeballs almost entirely subsided, as well as that of the lids, which have also lost all appearance of villosity. Bowels regular.—Cont. solutio. Half diet.

23d.—Discharged cured. Both eyes in a natural state.

Case II.—Richard Moore, 36th Regiment, æt. forty-five; admitted 19th March, 1822. Was admitted last night, affected with acute ophthalmia of both eyes, attended with great pain, sensation of sand, intolerance of light, and a great discharge of thick, white, puriform matter; the conjunctiva sclerotica so very vascular, that scarcely a white spot could be discovered, and some degree of chemosis around the cornea; the lids slightly villous, and very vascular. The inflammation is merely local, and there is no general excitement of the system. Instillat solutio arg. nit. gr. iv. ad aquæ ℥j. Low diet.

21st.—Pain in the eyes has entirely subsided; the vascularity diminished, and the chemosis subsided. Complains of only a slight sensation of sand.—Cont. ut antea. Low diet.

23d.—Feels the eyes free from pain or sensation of sand; intolerance of light and lachrymation considerably diminished, as also the puriform discharge and vascularity of the conjunctiva.—Cont. solutio. Half diet.

27th.—Intolerance of light and lachrymation entirely subsided, and vascularity diminishing daily; bowels regular.—Cont. sol. Half diet.

30th.—Has no complaints now, except some slight vascularity of the conjunctiva, and a little puriform discharge.—Cont. sol. Half diet.

April 3d.—Has no puriform discharge, and the vascularity has subsided.—Cont. solutio. Half diet.

7th.—Discharged cured.

Case III.—Charles Hole, 1st Royal Dragoons, æt. twenty-five; admitted 29th March, 1822, with acute inflammation of the left eye, attended with intolerance of light, lachrymation, pain, and sensation of sand in the eye; the conjunctiva sclerotica extremely vascular; the vessels of a bright red colour, running at right angles from each other, and forming the appearance of a net-work over the ball, except immediately around the cornea, where they form a circle of radiated minute vessels, lost in its circumference. Was a patient in No. 7 ward when he was first attacked with this inflammation, at about five o'clock yesterday morning, by a sensation in the organ as if something had been blown into it.—Low diet. Instillat solutio argent. nitrat. gr. iv. ad aquæ ℥j. bis in die oculo sinist.

30th.—Vascularity of eyeball considerably diminished; the pain entirely subsided, and the lachrymation and intolerance of light nearly so; bowels regular.—Cont. solutio. Low diet.

April 2d.—Intolerance of light and lachrymation entirely subsided, and the vascularity nearly so.—Cont. ut antea. Half diet.

4th.—Discharged cured.

Case IV.—William Sparks, 13th Foot, æt. twenty-nine; admitted 31st October, 1822. Affected with pain in both eyes, accompanied by great intolerance of light, lachrymation, and puriform discharge; the conjunctiva extremely vascular, and the lids villous: the complaint more severe in the right eye than in the left. This attack came on three days ago, and was occasioned by a severe wetting received during a review; had only joined his regiment three weeks, from the General Hospital at Dublin, where he was confined for eight months with severe ophthalmia; for which, he states, he was bled from the arms twenty-seven times, and that at each time he never lost less than two pounds, often more; fourteen of which bleedings took place in the first fortnight after his admission. He also states, that there were thirty-five blisters applied to the whole of his head; and, he is certain, upwards of fifty to his neck, back, and behind the ears. Bluestone was repeatedly applied, and frequently occasioned considerable swelling of the lids, and pain; and it was often used immediately after the large bleedings. He underwent two full courses of mercury. Some drops were employed, but he cannot tell what they were.—Instillatur solutio argent. nitrat. gr. iv. ad aquæ ℥j. amb. oculis bis in die. Low diet.

November 1st.—The pain in both eyes has much abated, and the intolerance of light is not so distressing. Bowels regular.—Cont. solutio.

2d.—Says he has little or no pain in the right eye, and that the left is entirely free from it. The intolerance of light and the lachrymation have much subsided, and there is scarcely any appearance of puriform discharge.—Cont. ut antea. Low diet.

4th.—Both eyes perfectly free from pain or any uneasiness, and the intolerance of light nearly subsided.—Cont. solutio.

6th.—Intolerance of light subsided, and the lachrymation and puriform discharge nearly so.—Cont. solutio. Low diet.

9th.—Vascularity of the eyeballs considerably diminished, and the puriform discharge and lachrymation subsided; bowels regular.—Cont. solutio. Low diet.

13th.—Vascularity continues to diminish, and the acute inflammation has entirely subsided these several days past: the disease appears to be now much in the same chronic state as when he arrived from Dublin. Lids villous and vascular.—Cont. ut antea. Half diet.

18th.—The vascularity of the lids diminished, and the villosity appears gradually to subside.—Cont. solutio. Half diet.

24th.—Villosity of the lids continues to diminish, and the eyeballs are consequently less vascular.—Cont. solutio. Half diet.

December 21st.—The villosity of the lids removed; only some slight increase of vascularity remaining; cornea clear, and conjunctiva sclerotica natural. Discharged, cured.

*Cases of Chronic Ophthalmia.* CASE V.—William Jenkins, 47th Regiment, æt. thirty; admitted 9th June, 1822, labouring under great opacity and vascularity of the cornea of both eyes, and highly granulated lids; vision so much impaired, that he cannot walk without the assistance of a stick, or some one to guide him; complains of a dull pain in the eyes, and intolerance of light, which obliges him to wear a green shade. This complaint first attacked him on the 1st of last month, during his voyage from Bombay, by a violent pain in the eyes, which was soon followed by a profuse discharge of puriform matter. He was treated by venesection *ad deliquium*, twice repeated; blisters to the forehead, temples, and neck, and Goulard lotion. He states, that he received no benefit from the bleedings; that the pain continued unabated, until the blisters were applied about ten or twelve days after the attack. Never had sore eyes before.—Instillatur solutio argent. nitrat. gr. iv. ad aquæ ℥j. bis in die. Half diet.

12th.—The solution agrees very well with his eyes: it produces a sensation of roughness, and a smarting pain, for about fifteen or twenty minutes after it is dropped in; which then subsides, and he feels the eyes more comfortable.—Cont. ut antea. Half diet.

20th.—Vascularity of the cornea diminished, and his vision improved. —Cont. solutio. Half diet.

July 2d.—The vascularity of the eyeballs and cornea considerably abated, and his vision so much improved, that he can now see to walk about with ease.—Cont. solutio. Half diet.

15th.—Granulations of the lids diminished, as also the vascularity and opacity of the cornea.—Cont. solutio. Half diet.

August 1st.—The granulations of the lids continue gradually to subside, as also the opacity and vascularity of the cornea.—Cont. ut antea. Half diet.

15th.—Cornea of right eye nearly transparent, and free from red vessels; that of the left still opaque, especially at the superior part.—Cont. solutio. Half diet.

25th.—The granulations continue to diminish, and become less vascular; the cornea are also improving.—Cont. solutio. Half diet.

September 5th.—The granulations are nearly removed; the right cornea is almost clear, and the left gradually becomes more so.—Cont. solutio. Half diet.

14th.—Cornea of right eye quite transparent. There is still some opacity at the superior margin of the left cornea, but it does not interfere with the axis of vision; lids nearly smooth.—Cont. solutio.

24th.—Discharged: vision perfect; both eyes natural.

Case VI.—Benjamin Callow, 81st Regiment, æt. forty-five; admitted 24th April, 1822, affected with opacity and vascularity of the cornea of both eyes, and great villosity and vascularity of the lids; the right eye considerably more diseased than the left, and he cannot see any object distinctly with it; suffers little or no pain, but has some intolerance of light, and lachrymation. These complaints are the sequelæ of an acute ophthalmia, with which he was attacked twelve months back, at Cork; which was treated by general and local blood-letting, purgatives, blisters, &c.—Instillat. solutio argent. nitrat. gr. iv. ad aquæ ℥j. bis in die. Half diet.

27th.—Vascularity of the eyelids diminished, as also the intolerance of light and lachrymation. Bowels regular.—Cont. ut antea. Half diet.

May 3d.—Intolerance of light and lachrymation nearly ceased, and the vascularity is much diminished.—Cont. solutio. Half diet.

10th.—The villosity of the lids continues to subside gradually, and the eyes to recover their natural appearance.—Cont. ut antea. Half diet.

20th.—Vascularity and opacity of both cornea diminishing, and also the villosity of the lids.—Cont. solutio. Half diet.

June 7th.—Left cornea free from vascularity, and nearly so from opacity; right improving.—Cont. solutio. Half diet.

10th.—Vascularity of right cornea nearly subsided, and the opacity also, except in one small spot, which appears to be a small cicatrix; lids nearly smooth.—Cont. solutio. Half diet.

23d.—Discharged. The lids of both eyes perfectly healthy; left eye natural, vision perfect; right eye the same, except a small cicatrix, which is situated opposite the outer margin of the pupil, and renders vision in that direction indistinct.

CASE VII.—Charles Alefounder, 3d Foot, æt. twenty-six; admitted 26th July, 1822, labouring under granular lids, with opaque and vascular cornea, attended with puriform discharge and intolerance of light: the latter symptom is very troublesome, and is the only uneasiness he experiences, as he suffers no pain. He states that this complaint commenced last December, with acute pain in both eyes; and that he has been ever since in his regimental hospital, under treatment, where he was bled, cupped, leeched, blistered, had a seton placed in his neck, and used a variety of drops and bluestone.—Instillat. solutio argent. nitrat. gr. iv. ad aquæ ℥j. bis in die, amb. oculis.

30th.—The drops have agreed very well with the eyes: they feel a little rough and painful after they have been applied, but the eyes are more comfortable afterwards. Bowels regular.—Cont. solutio. Half diet.

August 17th.—Vascularity of eyeballs and lids much abated, as well as the accompanying symptoms.—Cont. solutio. Half diet.

September 19th.—Discharged; both eyes quite well.

[To be continued.]

